# Whitebark Pine, Population Density, and Home-Range Size of Grizzly Bears in the Greater Yellowstone Ecosystem

**DOI:** 10.1371/journal.pone.0088160

**Published:** 2014-02-10

**Authors:** Daniel D. Bjornlie, Frank T. Van Manen, Michael R. Ebinger, Mark A. Haroldson, Daniel J. Thompson, Cecily M. Costello

**Affiliations:** 1 Large Carnivore Section, Wyoming Game and Fish Department, Lander, Wyoming, United States of America; 2 U.S. Geological Survey, Northern Rocky Mountain Science Center, Interagency Grizzly Bear Study Team, Bozeman, Montana, United States of America; 3 University of Montana, College of Forestry and Conservation, Missoula, Montana, United States of America; The Ohio State University, United States of America

## Abstract

Changes in life history traits of species can be an important indicator of potential factors influencing populations. For grizzly bears (*Ursus arctos*) in the Greater Yellowstone Ecosystem (GYE), recent decline of whitebark pine (WBP; *Pinus albicaulis*), an important fall food resource, has been paired with a slowing of population growth following two decades of robust population increase. These observations have raised questions whether resource decline or density-dependent processes may be associated with changes in population growth. Distinguishing these effects based on changes in demographic rates can be difficult. However, unlike the parallel demographic responses expected from both decreasing food availability and increasing population density, we hypothesized opposing behavioral responses of grizzly bears with regard to changes in home-range size. We used the dynamic changes in food resources and population density of grizzly bears as a natural experiment to examine hypotheses regarding these potentially competing influences on grizzly bear home-range size. We found that home-range size did not increase during the period of whitebark pine decline and was not related to proportion of whitebark pine in home ranges. However, female home-range size was negatively associated with an index of population density. Our data indicate that home-range size of grizzly bears in the GYE is not associated with availability of WBP, and, for female grizzly bears, increasing population density may constrain home-range size.

## Introduction

Following its listing as threatened under the Endangered Species Act in 1975, the grizzly bear (*Ursus arctos*) population in the Greater Yellowstone Ecosystem (GYE) grew from approximately 200–350 bears in the mid-1980s [1] to at least 600 in 2012 [2]. Annual population growth rates of 4–7% were estimated during the 1980s and 1990s [3], but growth rates slowed to approximately 0–2% during the 2000s [4]. By the late 1990s, some evidence for density-dependent effects on reproduction and survival, particularly within the core area of Yellowstone National Park, were noted [3], [5]. Subsequently, substantial decline of an important, high-elevation food source, whitebark pine (WBP; *Pinus albicaulis*) seeds, began in the early 2000s. This decline has been attributed to warming temperatures in alpine and montane systems of western North America, facilitating an irruption of native mountain pine beetles (*Dendroctonus ponderosae*) that has killed vast stands from New Mexico to Canada [6]. White pine blister rust (*Cronartium ribicola*) is also known to infect WBP in the GYE, however its contribution to tree mortality has been much lower than that of mountain pine beetles [7]. Regardless of cause, on monitoring transects established in the GYE, 27% of marked trees >1.4 m tall (all age classes) died during 2008–2013, 37% of tagged trees >10 cm and ≤30 cm in diameter, and 72% of trees ≥30 cm in diameter [7].

Decreasing food availability and increasing population density may have similar negative effects on survival and reproduction and it is possible that either or both of these factors have contributed to the recent slowing of population growth in the GYE grizzly bear population. However, it can be difficult to differentiate their effects in demographic analyses. Consequently, we designed a study that focused on another important facet of life history, home-range size, to explore which factor might be acting more strongly. Home-range size has been demonstrated to reflect a species’ response to population density or resource variability [8], [9], [10]. Unlike the parallel demographic responses expected from decreasing food availability and increasing density, we hypothesized that grizzly bears would have opposing behavioral responses in terms of their space use, with increasing home-range size in the face of food resource limitation and decreasing home-range size in response to increasing population density.

An inverse relationship between home-range size and food availability has been demonstrated in a number of studies involving solitary species with overlapping home ranges. These include experimental manipulations of rodent populations [11], [12] and field studies of other rodents and large mammals [13], [14], [15]. Among ursids, American black bears (*Ursus americanus*) [16], [17], [18], [19] and Asiatic black bears (*Ursus thibetanus*) [20] have been observed to increase home-range size in a response to drought conditions and lower food availability, such as mast failures. In comparisons among populations, a negative relationship between home-range size and habitat productivity was evident in brown bears [21], [22].

Experiments with rodents also demonstrated that home-range size was negatively associated with density [23] and with the instantaneous rate of increase [24]. In both studies, similar results were observed under varying food conditions, and the increased aggression and territorial defense observed at greater densities suggested the effect was attributable to intraspecific intruder pressure. In field studies, negative relationships between density and home-range size have been observed for black bears [25], brown bears [21], [26], and other solitary species with overlapping home ranges, such as roe deer (***Capreolus capreolus***) [8], [27] and wild boar (*Sus scrofa*) [28], mostly through competition for space.

We used the dynamic changes in potentially competing influences of density and food decline [29] on home-range size as a natural experiment to examine hypotheses regarding resources and demographics of grizzly bears in the GYE. Our experiment was based on comparison of individual home ranges [9] for periods preceding and following WBP decline. If resource availability is a greater determinant of home-range size than population density, we predicted that grizzly bear home ranges following WBP declines would increase in area as diets shift and individuals increase movements to obtain alternate food sources [30]. Alternatively, if population density is a greater determinant of space use, we expected an inverse relationship between density and home-range size as more bears compete for space. Because space allocation among females is generally less plastic than males due to differences in philopatry, we predicted a stronger effect of resource availability for males but a stronger effect of population density for females.

## Materials and Methods

### Ethics Statement

Member agencies of the Interagency Grizzly Bear Study Team radiomarked grizzly bears in the GYE for research and monitoring purposes. Grizzly bear capture and handling procedures used for this study were reviewed and approved by the Animal Care and Use Committee (IACUC #201201) of the U.S. Geological Survey and procedures conformed to the Animal Welfare Act and to U.S. Government principles for the utilization and care of vertebrate animals used in testing, research, and training. Captures were conducted under U.S. Fish and Wildlife Service Endangered Species Permit [Section (i) C and D of the grizzly bear 4(d) rule, 50 CFR17.40 (b)], with additional state research permits for Wyoming, Montana, and Idaho, and National Park Service research permits for Yellowstone and Grand Teton National Parks.

### Study Area

The study area encompassed approximately 50,000-km^2^ of occupied grizzly bear range in the GYE [31], including Yellowstone and Grand Teton National Parks, and adjacent federal, state, private, and tribal lands in Wyoming, Montana, and Idaho. It consists of the Yellowstone Plateau and 14 contiguous mountain ranges at or above 1,500 m elevation and contains the headwaters of the Missouri-Mississippi, Snake-Columbia, and Green-Colorado river systems. Additional study area details are described in Schwartz et al. [3].

### Capture and Telemetry

We used culvert traps or Aldrich leg-hold snares to capture bears [32], [33]. Trapping efforts occurred in both front- (road access) and backcountry (no road access) settings within and outside national parks and wilderness areas. With the exception of dependent offspring, we fitted captured grizzly bears with radio transmitters. Adults were collared with VHF transmitters (Telonics, Inc., Mesa, AZ), whereas independent subadults were instrumented with expandable collars [32], glue-on or ear tag transmitters. We used a biodegradable canvas spacer to ensure collar drop. We conducted approximately 2–4 flights per month from mid-April through late November to locate and monitor instrumented bears. The number of flights was reduced from late November through March when most bears were denned. Location data for individual bears excluded trap locations and included only 1 den location per year. We used only annual locations of grizzly bears that had never been captured and translocated in response to conflicts with humans.

### Data Analysis

**Home-range size.** We used aerial VHF telemetry locations from instrumented grizzly bears ≥2 years of age, with ≥1 location in June or earlier, ≥1 location in September, and ≥10 (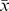 =  18.2, range  =  10–35) locations annually to estimate annual home ranges. Female cohorts included subadults (2–4 yrs), adults with cubs-of-the-year, and other adults (females with yearlings or 2-year-olds and lone adults). We excluded females with unknown reproductive status. Male cohorts were subadult (2–4 yrs) and adult. We log-transformed annual home-range estimates to represent a normal distribution.

We estimated annual home ranges with 95% *a*-local convex hull (LCH; [34] LoCoH.a in adehabitatHR for R v. 2.14.1, R Development Core Team, www.R-project.org) and 95% minimum convex polygon (MCP) techniques (ArcGIS v. 10.0, Environmental Systems Research Institute, Inc., Redlands, CA). For the LCH method, we used the maximum distance between any 2 locations in each bear’s annual data as the initial *a* value and then adjusted upward in increments of 0.5 (i.e., 1.5*a*, 2.0*a*, 2.5*a*, 3.0*a*). We graphed area versus *a* to choose the optimal value for our home ranges [34]. Whereas MCPs tend to overestimate home-range size [35], the LCH method reduces Type I error by excluding unused areas [36]. Because detection of changes in home-range size was important to test our hypotheses, we used LCH home ranges as our primary data source. We also used the 95% MCP estimates to evaluate the robustness of our findings by examining home ranges at a larger scale.

**WBP decline.** WBP in the GYE was mapped in 2009 with data from satellite imagery and historic vegetation maps and updated in 2010 to reflect new information obtained from recent satellite imagery and aerial reconnaissance [37]. This effort used data from land management agencies in the GYE and was reclassified to create a consistent geospatial layer of WBP distribution. The extent of mortality of WBP was estimated with the Landscape Assessment System (LAS) project, which used aerial transects and aerial photographic evidence to estimate and rank WBP mortality throughout the GYE [37], [38]. LAS rankings ranged from 0 to 6, with rankings ≥4 generally indicating no live overstory remaining. Because the LAS project was conducted in 2009 and the greatest mortality of mature WBP peaked around 2009 with a decreased mortality rate since [7], [39], we classified 1989–1999 home ranges as pre-impact and 2007–2012 home ranges as impacted, the latter period representing substantially diminished WBP stands. For home ranges during the impact period, we used LAS data to adjust the proportion of WBP in the home range to account for tree mortality. We assumed that areas mapped as WBP vegetation but with LAS rankings of ≥4 represented stands that provided little to no foraging value to grizzly bears, and thus censored those from the proportion of WBP available in the home range. We used Geospatial Modeling Environment [40] to calculate the proportion of WBP available within each grizzly bear’s annual home range.

**Grizzly bear population density.** We developed a spatially and temporally explicit index of population density by dividing the GYE into 14– × 14-km (196 km^2^) grid cells, approximately matching the size of annual female home ranges (1975–2001 data; Interagency Grizzly Bear Study Team, unpublished data). Using telemetry locations and life-history of captured bears, we created the density index by back- and forecasting lifetime home ranges to indicate the presence of individuals in the population through space and time. We restricted telemetry data to known-aged individuals of ≥2 years that were captured for research or management purposes during 1975–2012 (870 individual bears). For bears with known mortality, we forecasted ranges through the year of mortality. For bears with unknown fates, we applied sex-specific survival probabilities (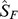 = 0.950, 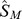 = 0.925; [3], [4]) to forecast the annual probability they remained in the population. We thus successively reduced the contribution of their annual ranges to the density index according to annual survival rates. We limited lifetime contribution of bears of unknown fates to a maximum age of 30 years [3]. Bears are long-lived, so the presence of unmarked bears may go undetected near the end of the sampling period because those bears are less likely to have been captured at the time of analysis. Because this would result in underestimation of the density index starting around 2007, we ended the density index in 2006 and used autoregression models ([41]; arima in stats for R v. 2.14.1, R Development Core Team, www.R-project.org) to project the density trend forward for 2007–2012 for each 14– × 14-km grid cell. We based those regressions on the previous 5 years of data (i.e., 2002–2006). Because we started with 1983 capture and telemetry records and our pre-impact period began in 1989, we did not need to correct for underestimation bias during the pre-impact period. We calculated the density index for each grid cell in a given year as the sum of proportional overlap of all lifetime activity ranges present during that year (Figure S1). We used Geospatial Modeling Environment [40] to calculate the density index values within each grizzly bear’s annual home range. A detailed description of the density index methods is provided in Appendix S1.

**Statistical analysis.** We tested our hypotheses by comparing a set of *a priori* models using Akaike’s Information Criterion (AIC_c_) [42]. We standardized independent, numeric variables to place variables on a common scale [43]. We used home-range size as the dependent variable in linear-mixed effects models (lme4 for R v. 2.14.1, R Development Core Team, www.R-project.org), with crossed random intercepts for year and individual bears. We evaluated the random effect structure for our models using restricted maximum likelihood estimation. Subsequently, we competed models with different fixed effects, but consistent random effect terms, using unrestricted maximum likelihood estimation. Because our inference was at the population level and inclusion of the random effects served to model correlation of responses within groups (bears or years), our models are equivalent to linear models with correlated errors; accordingly, we used marginal AIC_c_ (mAIC_c_), or simply AIC_c_, to test our hypotheses [44]. We analyzed females and males separately because of differences in home-range size [45]. For our model set we used combinations of 4 independent variables and their interactions: cohort, period (pre- and during WBP impact), proportion of WBP in home range, and grizzly bear density in home range. Our main treatment was based on the 2 periods and interactions with indices of WBP availability and grizzly bear population density; we used these interactions to test if the relationship between home-range size and population density or home-range size and WBP would be different during the 1989–1999 and 2007–2012 periods. Because the WBP and density indices were spatially and temporally explicit, we were also able to test spatial relationships of the two indices with home-range size of individuals, regardless of period. We assessed the importance of model terms by comparing model-averaged beta estimates and associated 95% confidence intervals. Home-range estimates may be influenced by sample size, so we included the number of locations as a variable in each model to account for this. We also included the number of locations as a single variable in a separate model to test whether it was more influential than other covariates. We examined relative importance of the relationship with home-range size for the density index versus WBP by calculating the cumulative AIC_c_ weights of models that included each respective variable (model set was balanced, with each variable occurring in 6 models with the same combination of covariates). Data used in our analyses are provided in Appendix S2.

## Results

**Figure 1:**
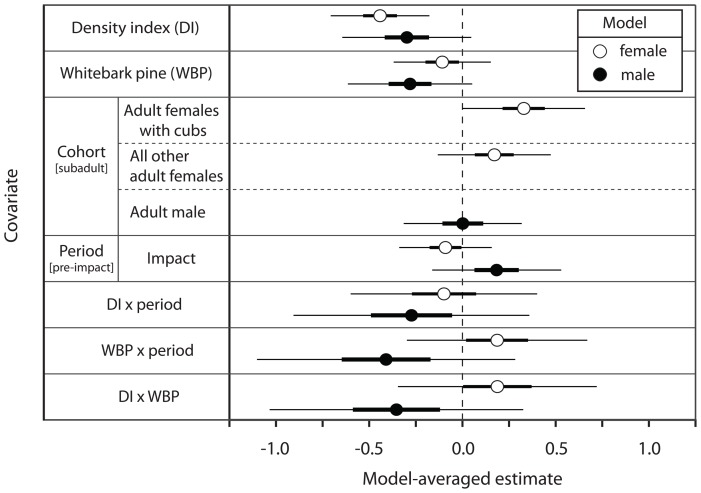
Beta estimates for covariate effects on home-range size of female and male grizzly bears. Model-averaged beta estimates from linear mixed-effects models of cohort, period (pre-whitebark pine [WBP] impact [1989–1999] and WBP impact [2007–2012]), density index (DI), and proportion of WBP in home range (adjusted for mortality during impact period**)** on home-range size (km^2^; 95% *a*-local convex hull) of female (open circles) and male (gray circles) grizzly bears in the Greater Yellowstone Ecosystem. Reference groups for cohort and period are in brackets. We applied z-score transformations to WBP and DI.

**Figure 2:**
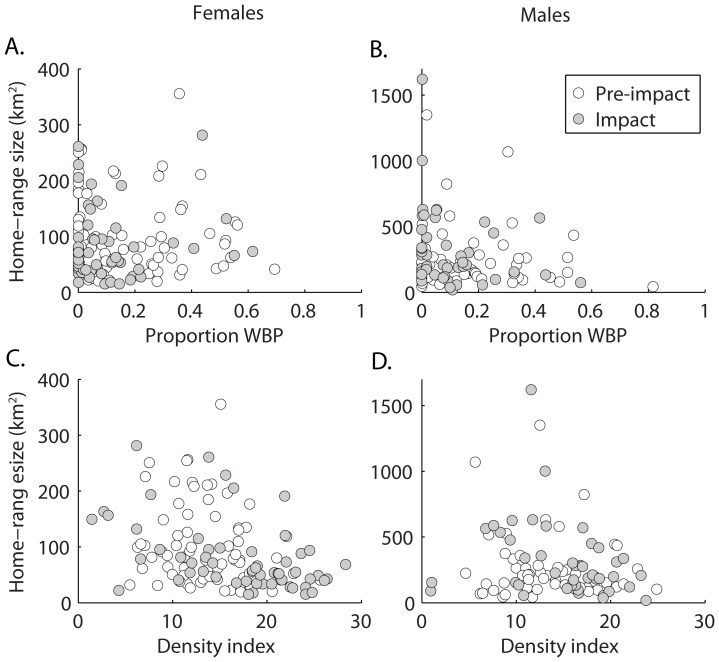
Relationships between home-range size and whitebark pine (A, B) or density (C, D). Relationships between home-range size of female (A, C) and male (B, D) grizzly bears (95% *a*-local convex hull), proportion of whitebark pine (WBP) or density index within home ranges, and period before (1989–1999; open circles) and during (2007–2012; gray circles) impact of WBP decline in the Greater Yellowstone Ecosystem.

We analyzed 223 annual home ranges of 147 individual grizzly bears, with 51 home ranges containing <1% WBP. Home-range size of females decreased from the pre-impact to impact period but did not change among males (Table 1). The proportion of WBP in home ranges declined from the pre-impact to impact period for females and males, whereas density increased, particularly among females (Table 1). The random intercept term for individual bears ranged from 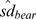 = 0.38 to 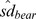 = 0.54 for the four dataset combinations based on sex and home-range metric (MCP and LCH) for our most saturated model. The inter-class correlations of home-range size within individuals (i.e., clusters) varied from 0.35 to 0.50. For male and female grizzly bears we observed little to no support for relationships between home-range size and cohort or period (Tables 2 and 3, Figure 1). For females, there was no support for a relationship between home-range size and WBP availability for either home-range metric (LCH: 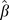 = –0.109, 95% CI = –0.370 to 0.152; Table 2, Figure 1; MCP: 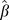 = –0.128, 95% CI = –0.389 to 0.132; Table 4, Figures 1 and 2). However, the density index was negatively associated with female home-range size, regardless of period, for LCH (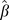 = –0.443, 95% CI = –0.708 to –0.178, *R^2^* = 0.14; Table 2, Figures 1 and 2) and MCP (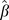 = –0.465, 95% CI = –0.710 to –0.219, *R^2^* = 0.15; Table 4). For male LCH home ranges, WBP and density models ranked high (Table 3), but confidence intervals of their model-averaged beta estimates overlapped zero, thus lacking distinct evidence of an association with size of home range (WBP: 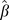 = –0.284, 95% CI = –0.618 to 0.050; density index: 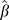 = –0.301, 95% CI = –0.648 to 0.045; Figures 1 and 2). For male MCP home ranges, however, we detected a negative relationship between density index and home-range size (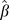 = –0.445, 95% CI = –0.859 to –0.032; Table 5), but not for WBP (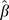 = –0.299, 95% CI = –0.738 to 0.139; Table 5). The relative importance of the association with female home-range size was distinctly greater for density index (LCH: cumulative AIC_c_ weight [Σ*w_i_*]  = 1.00; MCP: Σ*w_i_* = 1.00) than for WBP (LCH: Σ*w_i_* = 0.13; MCP: Σ*w_i_* = 0.06). For males, the relative importance of density index (LCH: Σ*w_i_* = 0.61; MCP: Σ*w_i_* = 0.89) was moderate over WBP (LCH: Σ*w_i_* = 0.55; MCP: Σ*w_i_* = 0.54). For both male and female grizzly bear home ranges, support for the model with only number of locations was much less than their respective top models, except for male LCH ranges (female LCH: 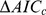 = 10.4, male LCH: 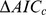 = 0.8; female MCP: 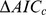 = 10.9, male MCP: 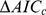 = 4.7).

**Table 1:** Metrics^1^ of home-range size^2^, proportion whitebark pine (WBP; adjusted for mortality during impact period), and density index associated with female and male grizzly bear home ranges in the Greater Yellowstone Ecosystem during pre-WBP impact (1989–1999) and WBP impact (2007–2012) periods.

	Metric	Pre-Impact	Impact	*t*-test^3^
Females	*n*	71	56	
	Home-range size (km^2^)	103.2±71.8	80.9±62.7	*t* = 2.05, *p* = 0.043
	WBP proportion	0.19±0.18	0.11±0.15	*t* = 2.78, *p* = 0.006
	Density index	13.58±4.22	16.99±6.75	*t* = –3.31, *p* = 0.001
Males	*n*	51	45	
	Home-range size (km^2^)	267.9±253.3	308.9±288.6	*t* = –0.56, *p* = 0.580
	WBP proportion	0.19±0.18	0.10±0.13	*t* = 2.90, *p* = 0.005
	Density index	13.18±4.79	15.01±5.21	*t* = –1.78, *p* = 0.078

1 Home-range, WBP, and density values are
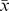
± 1 sd.

2 Home-range size based on 95% *a*-local convex hull.

3 Test results based on log transformation of home-range size and z-score transformation of WBP proportion and density index.

**Table 2:** Akaike’s Information criteria (AIC_c_) of linear mixed-effects models^1^ to examine relationships of covariates^2^ with home-range size (km^2^; 95% *a*-local convex hull) of female grizzly bears in the Greater Yellowstone Ecosystem.

Model	AIC_c_	ΔAIC_c_	AIC_c_ weight
DI	249.62	0.00	0.62
Cohort, period, DI	252.01	2.38	0.19
Cohort, period, DI, WBP	253.68	4.06	0.08
Cohort, period, DI, DI×period	254.19	4.57	0.06
Cohort, period, DI, WBP, DI×WBP	255.61	5.99	0.03
Cohort, period, DI, WBP, DI×period, WBP×period	257.83	8.21	0.01
WBP	262.16	12.54	0.00
Cohort, period, WBP	262.71	13.09	0.00
Cohort, period, WBP, WBP×period	264.43	14.80	0.00

1 Results based on log transformation of home-range size and z-score transformation of WBP and DI.

2 Covariates: period  =  pre-impact (1989–1999) and impact (2007–2012) periods, cohort  =  age class, DI  =  density index, WBP  =  proportion of whitebark pine in home range adjusted for tree mortality during the impact period.

Year and individual bear were included as random effects. The number of locations used to create each home range was included in all models. Density index (DI) was the only covariate for which the 95% confidence interval for the model-averaged beta estimate did not overlap zero.

**Table 3:** Akaike’s Information criteria (AIC_c_) of linear mixed-effects models^1^ to examine relationships of covariates^2^ with home-range size (km^2^; 95% *a*-local convex hull) of male grizzly bears in the Greater Yellowstone Ecosystem.

Model	AIC_c_	ΔAIC_c_	AIC_c_ weight
WBP	229.19	0.00	0.34
DI	229.28	0.09	0.32
Cohort, period, DI, WBP	231.76	2.56	0.09
Cohort, period, DI	231.81	2.61	0.09
Cohort, period, DI, WBP, DI×WBP	233.27	4.08	0.04
Cohort, period, WBP	233.54	4.34	0.04
Cohort, period, DI, DI×period	233.79	4.59	0.03
Cohort, period, DI, WBP, DI×period, WBP×period	234.19	5.00	0.03
Cohort, period, WBP, WBP×period	235.17	5.97	0.02

1 Results based on log transformation of home-range size and z-score transformation of WBP and DI.

2 Covariates: period  =  pre-impact (1989–1999) and impact (2007–2012) periods, cohort  =  age class, DI  =  density index, WBP  =  proportion of whitebark pine in home range adjusted for tree mortality during the impact period.

Year and individual bear were included as random effects. The number of locations used to create each home range was included in all models. The 95% confidence intervals for the model-averaged beta estimates of all covariates overlapped zero.

**Table 4:** Akaike’s Information criteria (AIC_c_) of linear mixed-effects model^1^ to examine relationships of covariates^2^ with home-range size (km^2^; minimum convex polygon) of female grizzly bears in the Greater Yellowstone Ecosystem.

Model	AIC_c_	ΔAIC_c_	AIC_c_ weight
DI	249.61	0.00	0.86
Cohort, period, DI	254.86	5.25	0.06
Cohort, period, DI, WBP	256.19	6.58	0.03
Cohort, period, DI, DI×period	257.10	7.50	0.02
Cohort, period, DI, WBP, DI×WBP	257.34	7.73	0.02
Cohort, period, DI, WBP, DI×period, WBP×period	260.75	11.14	0.00
WBP	262.43	12.82	0.00
Cohort, period, WBP	267.45	17.85	0.00
Cohort, period, WBP, WBP×period	269.36	19.75	0.00

1 Results based on log transformation of home-range size and z-score transformation of WBP and DI.

2 Covariates: period  =  pre-impact (1989–1999) and impact (2007–2012) periods, cohort  =  age class, DI  =  density index, WBP  =  proportion of whitebark pine in home range adjusted for tree mortality during the impact period.

Year and individual bear were included as random effects. The number of locations used to create each home range was included in all models. Density index (DI) was the only covariate for which the 95% confidence interval for the model-averaged beta estimate did not overlap zero.

**Table 5:** Akaike’s Information criteria (AIC_c_) of linear mixed-effects model^1^ to examine relationships of covariates^2^ with home-range size (km^2^; minimum convex polygon) of male grizzly bears in the Greater Yellowstone Ecosystem.

Model	AIC_c_	ΔAIC_c_	AIC_c_ weight
DI	228.17	0.00	0.33
Cohort, period, DI, WBP	229.08	0.91	0.21
Cohort, period, DI, WBP, DI×period, WBP×period	229.70	1.53	0.15
WBP	230.73	2.55	0.09
Cohort, period, DI	230.87	2.69	0.08
Cohort, period, DI, WBP, DI×WBP	231.02	2.85	0.08
Cohort, period, DI, DI×period	232.14	3.97	0.04
Cohort, period, WBP	235.18	7.01	0.01
Cohort, period, WBP, WBP×period	236.18	8.01	0.01

1 Results based on log transformation of home-range size and z-score transformation of WBP and DI.

2 Covariates: period  =  pre-impact (1989–1999) and impact (2007–2012) periods, cohort  =  age class, DI  =  density index, WBP  =  proportion of whitebark pine in home range adjusted for tree mortality during the impact period.

Year and individual bear were included as random effects. The number of locations used to create each home range was included in all models. Density index (DI) was the only covariate for which the 95% confidence interval for the model-averaged beta estimate did not overlap zero.

Given the relationship between home-range size and density among females, we explored whether this relationship would be non-linear, with home-range size declining more rapidly as a threshold density is reached. We tested a post-hoc model by adding the square root of density to the female models but only found moderate support for this hypothesis (LCH: 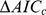 = 2.10; MCP: 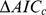 = 2.09).

## Discussion

Our findings suggest that for female grizzly bears in the GYE, home-range size is more strongly associated with population density than with availability of WBP. Our cumulative model weights for each covariate support this interpretation. Importantly, lack of support for a density × period interaction indicates that where bear densities are high, female home ranges tend to be smaller regardless of time period (i.e., this relationship was apparent even during 1989–1999, when overall bear densities were lower compared with 2007–2012). Additionally, the lack of a density × WBP interaction suggests the relationship between home-range size and density was independent of proportion of WBP in home ranges.

The association between population density and female home-range size is not surprising considering their philopatric dispersal and overlapping matrilineal home ranges [21], [45]. In contrast, males may be less affected by changes in density or WBP due to their large home ranges, which allow them to maximize breeding potential and access to high-quality food resources (e.g., ungulate carcasses). Our findings suggest that males may have greater opportunities to accommodate effects of increasing densities and resource variation without adjusting home-range area. Moreover, subadult males often disperse great distances and can potentially find low-density areas in which to establish [26]. This may be why we only observed a population density effect among males for MCP home ranges, which were consistently larger (pre-impact period [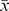
± 1 sd]: 431±392 km^2^; impact period: 524±527 km^2^) than LCH home ranges (Table 1). Although male home ranges did not show a temporal change between the two periods (LCH: Table 1; MCP: *t* = –0.96, *p* = 0.34), we had moderate evidence for an inverse relationship of MCP home-range size and density index at a spatial level, regardless of period. For male LCH home ranges, only the number of locations showed a statistical relationship with home-range size (neither density index nor WBP showed association). We speculate that sample sizes were insufficient to obtain reliable estimates of male LCH home ranges to detect relationships with density or WBP. Although much variation in home-range size remained unexplained because many factors not evaluated in this study contribute to variation in home-range size and home ranges are inherently variable among individuals [46], our results indicate greater influence of grizzly bear population density versus WBP resources.

Variability of grizzly bear foods in the GYE is high, and the natural masting cycle of WBP and WBP mortality since the early 2000s are particularly representative of this variation [47], [48]. Yet, our findings did not reveal a relationship between declining WBP in the GYE and home-range sizes of grizzly bears, nor was availability of WBP associated with the size of home ranges in general. However, female home-range size decreased with increasing population density, possibly through effects of competition for available space and avoidance behavior on foraging ability as individuals saturate quality habitats [23], [24], [25]. Thus, in areas where grizzly bear density is high, home-range size of female grizzly bears may be constrained due to intra-specific interactions [49].

## Supporting Information

Figure S1 Changes in an index of grizzly bear population density in the Greater Yellowstone Ecosystem, 1983–2012. Relative grizzly bear population density in 1983 (A), 1993 (B), 2003 (C), and 2012 (D). Yellowstone National Park (inner black line) and the grizzly bear Recovery Zone (outer blue line) are represented on each panel for reference.
(TIF)

Appendix S1 Methods to develop a spatially-explicit index of population density for grizzly bears in the Greater Yellowstone Ecosystem.
(PDF)

Appendix S2 Data used in analyses of home-range size of grizzly bears in the Greater Yellowstone Ecosystem.
(XLSX)
