# Supplementary figures and images for: Whitebark Pine, Population Density, and Home-Range Size of Grizzly Bears in the Greater Yellowstone Ecosystem
Source: PLoS One. 2014 Feb 10;9(2):e88160. doi: 10.1371/journal.pone.0088160 (PMC3919729; doi:10.1371/journal.pone.0088160)

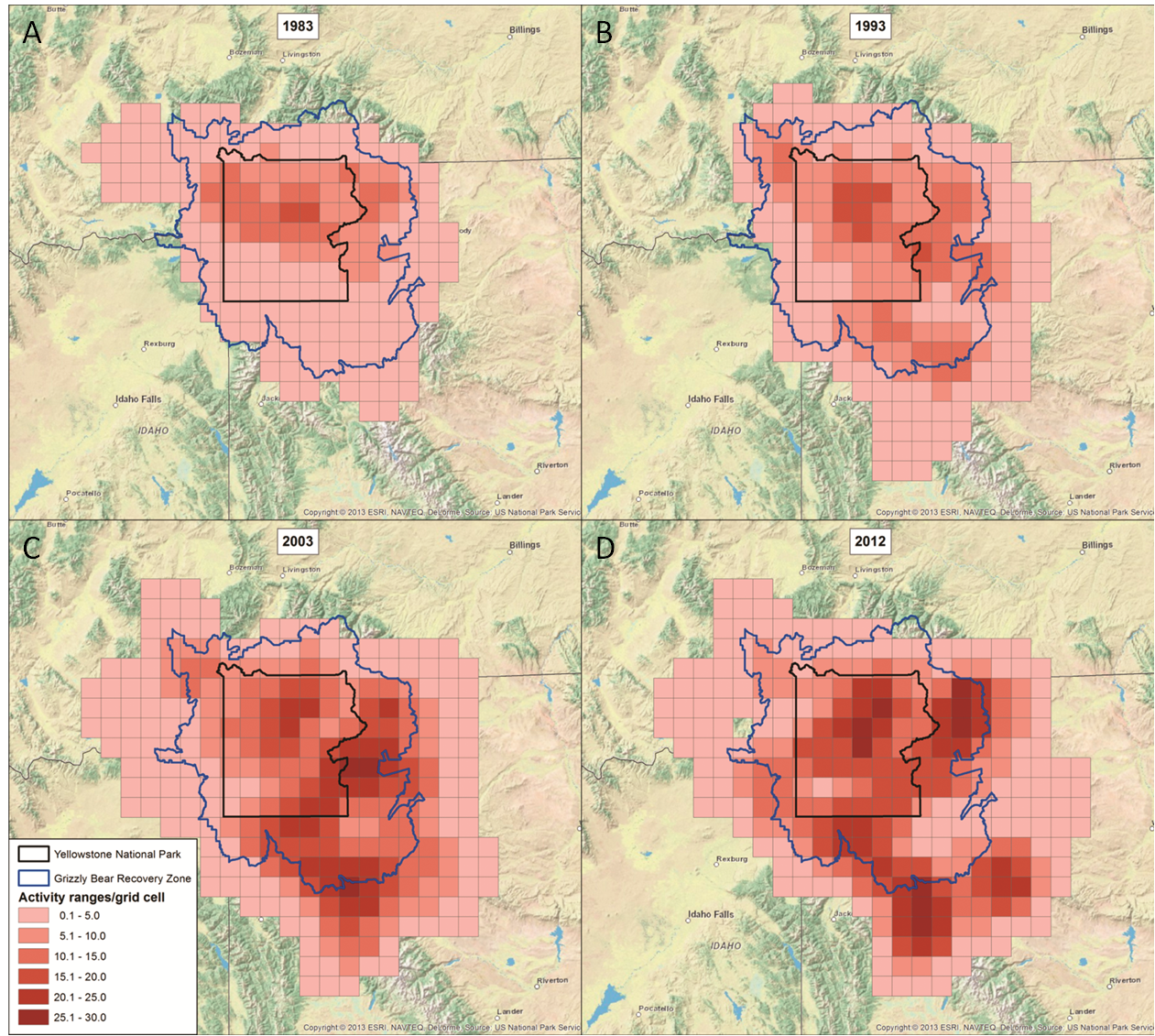

Supplement: Figure S1 — Changes in an index of grizzly bear population density in the Greater Yellowstone Ecosystem, 1983–2012. Relative grizzly bear population density in 1983 (A), 1993 (B), 2003 (C), and 2012 (D). Yellowstone National Park (inner black line) and the grizzly bear Recovery Zone (outer blue line) are represented on each panel for reference. (TIF) [file pone.0088160.s001.tif]
