# Supplementary material for: Whitebark Pine, Population Density, and Home-Range Size of Grizzly Bears in the Greater Yellowstone Ecosystem
Source: PLoS One. 2014 Feb 10;9(2):e88160. doi: 10.1371/journal.pone.0088160 (PMC3919729; doi:10.1371/journal.pone.0088160)
Supplement: Appendix S1 — Methods to develop a spatially-explicit index of population density for grizzly bears in the Greater Yellowstone Ecosystem. (PDF) [file pone.0088160.s002.pdf]

Appendix SA. *A Broad-Scale Spatially Explicit Annual Population Density Index for Grizzly Bears in the Greater Yellowstone Ecosystem: Synthesizing Life History, VHF telemetry, and Capture Data.*

INTRODUCTION

Although monitoring protocols for grizzly bears in the Greater Yellowstone Ecosystem (GYE) were not designed for density estimation, efforts to capture and radio-monitor individuals for research and management purposes began in 2975 and have been intensive and consistent since the early 1980s, involving over 1,800 captures of 870 individual bears, with capture effort distributed throughout the GYE. Estimating actual population density is unnecessary for most ecological questions concerning processes sensitive to the number of individuals involved and in most cases a reliable index is suitable for exploration of the importance of density dependence [1]. Accordingly, we developed a density index for grizzly bears in the GYE using a combination of very high frequency (VHF) telemetry data and histories (i.e., dates of capture, annual reproduction, cast transmitters, and known mortalities) of individual bears from 1983 through 2012. Using an annual time step and a coarse spatial scale (14 km × 14 km) approximating the average female grizzly bear activity range, we constructed a spatio-temporally explicit index of grizzly bear densities allowing exploration of the relative contribution of grizzly bear density in relation to variation in appropriate response variables (e.g., home-range size, survival, and movement parameters).

METHODS

The long-lived nature of grizzly bears, coupled with the substantial and sustained research efforts over time in the GYE has resulted in an accumulation of longitudinal data that present a unique opportunity to quantify the presence of individuals over time in discrete spatial units. Spatially, our approach was based on using locational data (VHF and capture locations) to reconstruct individual bears' extent of use, which we represent with a single, lifetime activity range. Temporally, we extruded these 2-dimensonal ranges through time from the age of independence (≥2 years of age) through the known or estimated year of death. We restricted telemetry data to known-aged individuals captured for research or management purposes during 1983–2012 and explicitly accounted for bears transported for management purposes.

Lifetime Activity Ranges

Spatial component.--We selected a circular activity range as the spatial metric for grizzly bear ranges. We generated lifetime activity ranges for all individuals assuming they were similar among cohorts within sexes. We used a sex-specific mean activity radius (γ_{sex}) and individuals' lifetime center of activity $\left(\varphi_{x,y}\right)$ to define the lifetime activity range. We used the harmonic mean of all VHF locations as our estimate of an individual's center of activity ($\hat{\varphi}_{x,y}$). For individuals located only once in their lifetime (i.e., captured once), we used the capture location to estimate the center of activity. We calculated the activity radius as follows:

$$\gamma_{sex} = \left(x - \varphi_x\right)^2 + \left(y - \varphi_y\right)^2$$

To reduce bias in calculating the sex-specific activity radii we restricted data to bear-years containing ≥5 unique months of data during May–December, in which at least 1 VHF location was recorded for each unique month. For bears that were transported during their lifetime, we included VHF locations up to the time of first transport. For individuals that were transported, we evaluated if the transport was successful or if the bear returned to its natal range. For bears that established new ranges, we created a second (temporally non-overlapping) activity range. To avoid overestimating bear ranges, we used the 80th percentile of distances between locations from the center of activity, from which we calculated the mean lifetime activity radius for each bear. The sex-specific, mean radii were γ_{male} = 24.3 km γ_{female} = 12.8 km (Figure S1).

Temporal component.--To account for changes in density over time, we extruded the spatial activity ranges through the time dimension according to the lifespan of individuals. Age at capture of each bear was determined from cementum annuli (Matson's Laboratory LLC, Missoula, Montana, USA) from which we estimated the birth year. We did not include cub and yearling contributions to density. Accordingly, activity ranges were hind-cast from the year of capture to the year the bear was age 2. For bears with known mortality, we forecasted activity ranges through the year of mortality, discretizing the lifetime activity range into annual ranges. This can be visualized as a cylinder where the base is the spatial extent of the lifetime activity range and the height represents time from age of independence (≥2 years old) to death (Figure S2A). For bears whose fates were unknown, we used sex-specific survival probabilities (S_F = 0.950, S_M = 0.925; [2,3]) to forecast the annual probability they remained in the population. We

thus reduced the contribution of their annual ranges to the density index according to annual survival rates. For these bears with unknown fates we limited their lifetime contribution to a maximum age of 30 years (Figure S2B).

Density Index Calculation

The spatial resolution of our density index was based on 196-km^2 square grid cells, which approximates the annual home-range size (approximately 200 km^2) of female bears (Interagency Grizzly Bear Study Team [IGBST], unpublished data) and the extent (448 km × 420 km; i.e., a 32 × 30 sampling frame of 960 grid cells) was well beyond known grizzly bear distribution ([4]). We calculated the density index on an annual basis for each grid cell. Specifically, we determined the proportion of each cell covered by individual activity ranges. Cells that were completely covered by an activity range received a value of 1 for an individual bear. For cells partially covered by an activity range, the contribution to the density index was based on its proportional coverage of the grid cell (Figure S3A). We calculated the density index for each grid cell in a given year as the sum of proportional overlap of all lifetime activity ranges present during that year (Figure S3B).

Our approach of hind-casting and forecasting activity ranges through time has several important caveats. First, the density index is based on bears that were captured and thus may be biased because of trap effort. Therefore, as part of our evaluation, we tested the strength of this relationship. Second, the initial and ending years of the 1975–2012 range underestimate densities relative to the central years of this period. This is unavoidable, as we could not include bears that would have been trapped in the past and forecast into the future (prior to 1983) or, alternatively, bears captured in the future and back-cast to the present (post 2012). Our use of the density index starts in 1983, which substantially reduces this bias for the initial years because trapping occurred during 1975–1983. However, there were no future captures from which to back-cast to inform the later years of the density index, resulting in underestimation of the density index starting around 2007. Therefore, we ended the density index in 2006 and used ARIMA(1,1,1) forecasting accounting for drift (i.e., $d > 0$) based on the previous 5 years of density information (2002–2006) to project trends forward to 2012 on a cell-by-cell basis (i.e., 960 time-series projections).

Model Evaluation

Trapping effort diagnostics.--To determine whether the density index was biased as a function of where cumulative trapping effort was greatest, we evaluated that potential impact on the density index for a 16-year period (1996–2011) for which detailed trapping records (i.e., grizzly captures/trap night) existed. Because of large home ranges of grizzly bears, they are often captured some distance from their center of activity. To account for this, we calculated the mean distance from individual bear activity centers to the locations where they were trapped for all non-transport influenced bears ($\bar{x}$ = 14.5 km) and used this value as a radius to define an "area of influence" for each trap site. We then calculated annual trapping effort based on the total number of trap-nights/site/year, similar to how we calculated the grizzly bear density index on an annual, cell-by-cell basis. Unlike the density index, the trapping index was calculated as the cumulative trapping effort for each year in the assessment period (1996–2011) to give an estimate of total trapping effort for each grid cell.

At the grid-cell level the cumulative trap effort is, by definition, restricted to being non-negative over time. Combined with an increasing grizzly bear population [2,3] during the evaluation period, we expected a positive correlation between the indices if we were to evaluate the relationship annually as a single correlation of the full grid extent. To avoid this confounding relationship, we instead focused on the correlation of the two time-series within each grid cell during 1996–2011.

Within each grid cell we evaluated the correlation between the first derivatives of trapping effort and bear density with respect to time. In other words, by examining the "detrended" time-series, we evaluated whether there was a correlation in the way the indices changed over the time period for each grid cell. The extent of the study grid resulted in a large proportion of cells having both zero density and zero trapping effort. These cells are uninformative and therefore were excluded from the trapping effort diagnostics. Furthermore, 29% of the cells with non-zero cumulative density values for the time series were cells where there was no trapping influence (i.e., trapping index = 0) for the entire time period. These cells were not solvable in terms of correlations due to the constant nature of the trapping time series and were excluded from the summary. Of the remaining cells (n = 250), there was little correlation between the rate of change of the cumulative trapping index and that of the density index ($\bar{x} = 0.07;\ \sigma = 0.28;$ Figure S4).

Flight observation validation.--We quasi-validated the density index using an independent sample providing information on geographic variation in grizzly bear density. Since the 1970s, the IGBST has used aerial surveys to collect observations of grizzly bears, particularly females with cubs-of-the-year. Before 1997, observations flights were conducted within established bear management units. In 1997, some of the larger units were split and several units were added outside of the previously-established units. These new units are known as bear observation areas (BOAs), and data from 28 of these 37 BOAs have been consistently collected since 1998. The sampling area of 9 BOAs on the eastern side of the GYE, where bears feed on army cutworm moths (*Euxoa auxiliaris*), expanded eastward over the study period, therefore these units were excluded from analyses. Typically, 2 observation flights are conducted each year within each BOA, without the aid of radio-telemetry. Flight duration, number of total bears observed, and number of bear groups are recorded for each flight. Single independent bears were considered a group, as were females with offspring. We used counts of bear groups, instead of individual bears, because our density index was based on individuals ≥2 years old.

We compared bear observation data with our density index for 1998–2006, with BOAs as the sampling unit (Figure S5). Our grid cells for the density index were 196 km^2, whereas BOAs were larger, ranging from 336 km^2 to 1,697 km^2. Flight observation data were variable among years. During 1998–2006, annual observation rates ranged from 0 to 5.1 bear groups/hr/1,000 km^2. The data were positively skewed, with 40% of observations representing zero counts. At least one annual count of 0 was observed among 23 of 28 BOAs (82%). Because of this skewed distribution, we used the log-transformed observation rates (groups/hr/1,000 km^2 + 1) for analysis (Figure S5). Flight observations data were not particularly valuable for assessing a temporal trend in density, except for showing a slight positive increase over time when BOA data were pooled. However, calculating median observation rates during 1998–2006 within each BOA allowed us to document geographic variation in the density index. We summarized annual mean density indices across BOAs by weighting the value for each density unit based on its area of overlap within the BOA. We then summarized annual flight observation and density index data for each BOA and used Pearson's correlation coefficient to test if bear observation rates were positively associated with the density index. For the period 1998–2006, the mean density index was positively correlated with mean log-transformed counts of bear groups/hr/1,000 km^2 within BOAs (r = 0.725, P < 0.001, n = 28; Figure S6).

DISCUSSION

A valid population level density index is one that is directly related to density in such a way that it tracks temporal and spatial changes in density [1]. Our approach leveraged multiple datasets (capture, life history, and VHF telemetry) on 870 bears to reconstruct the spatial and temporal variability of bear occupancy at a coarse, yet meaningful, spatial scale to grizzly bear space use. Although trapping is the driving force for initiating inclusion in the dataset, the long-lived nature and large home ranges prevents the density index from simply being a reflection of cumulative trapping intensity in a given grid cell. In fact, where trapping information is available (post 1995) approximately 1/3 of the grid cells show positive density values yet have zero trapping influence, highlighting the mobility and large ranges of grizzly bears. Although data were not available for true external validation, our quasi-validation using aerial flight observation data suggests that our index tracks the temporal and spatial changes in population density. We conclude this index of population density is useful as a covariate in analyses that explore various aspects of grizzly bear ecology.

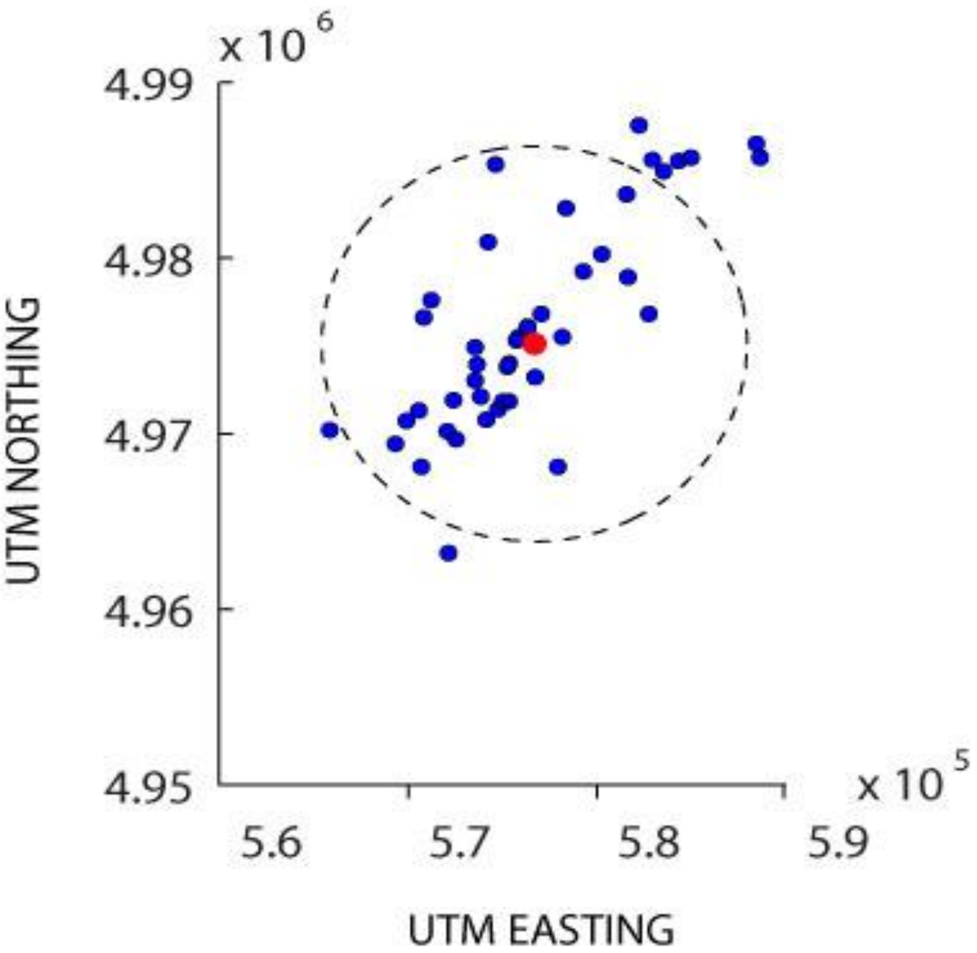

Figure S1. Example of lifetime grizzly bear activity range. Red dot indicates the harmonic center of activity, blue dots are VHF locations, and the dashed line is the 80th percentile of distances of all VHF locations to the activity center.

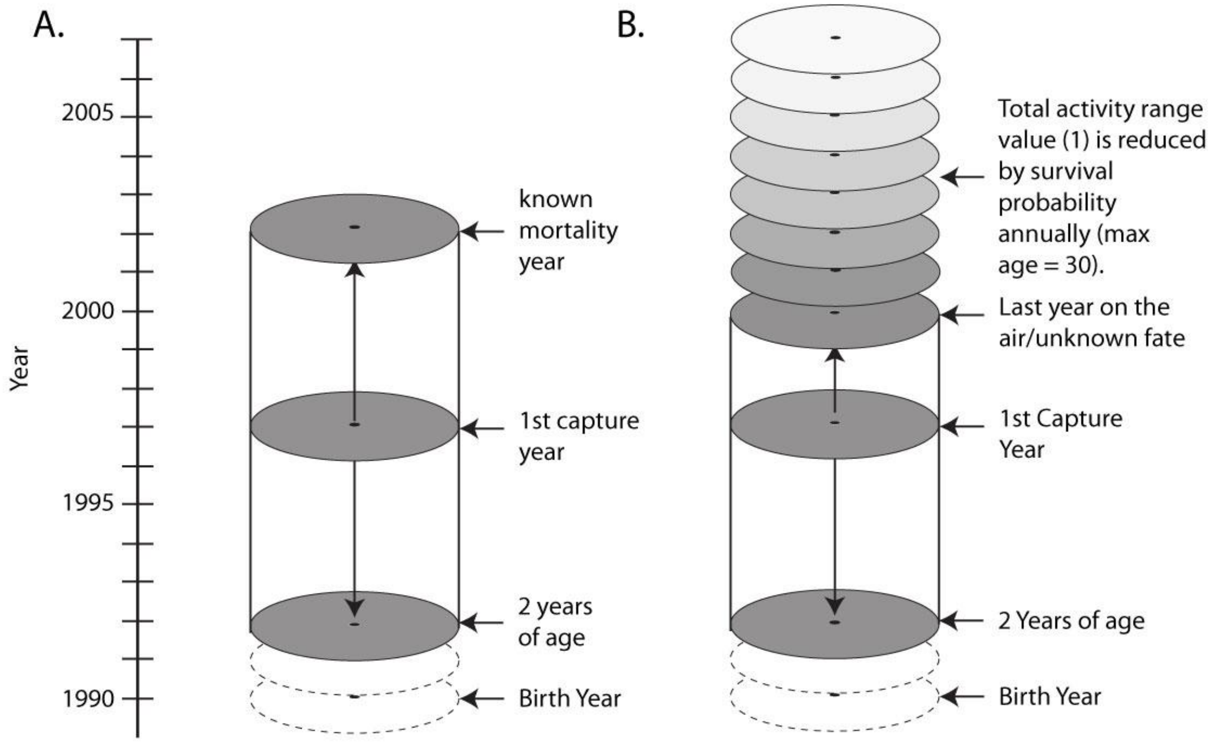

Figure S2. Lifetime grizzly bear activity range estimation. A 3-dimensional view showing how lifetime activity ranges are created for grizzly bears with (A) known and (B) unknown fates. Once the age at capture is determined, the lifetime activity area is created by hindcasting to age 2 and forecasting until death (A) or age 30 (B).

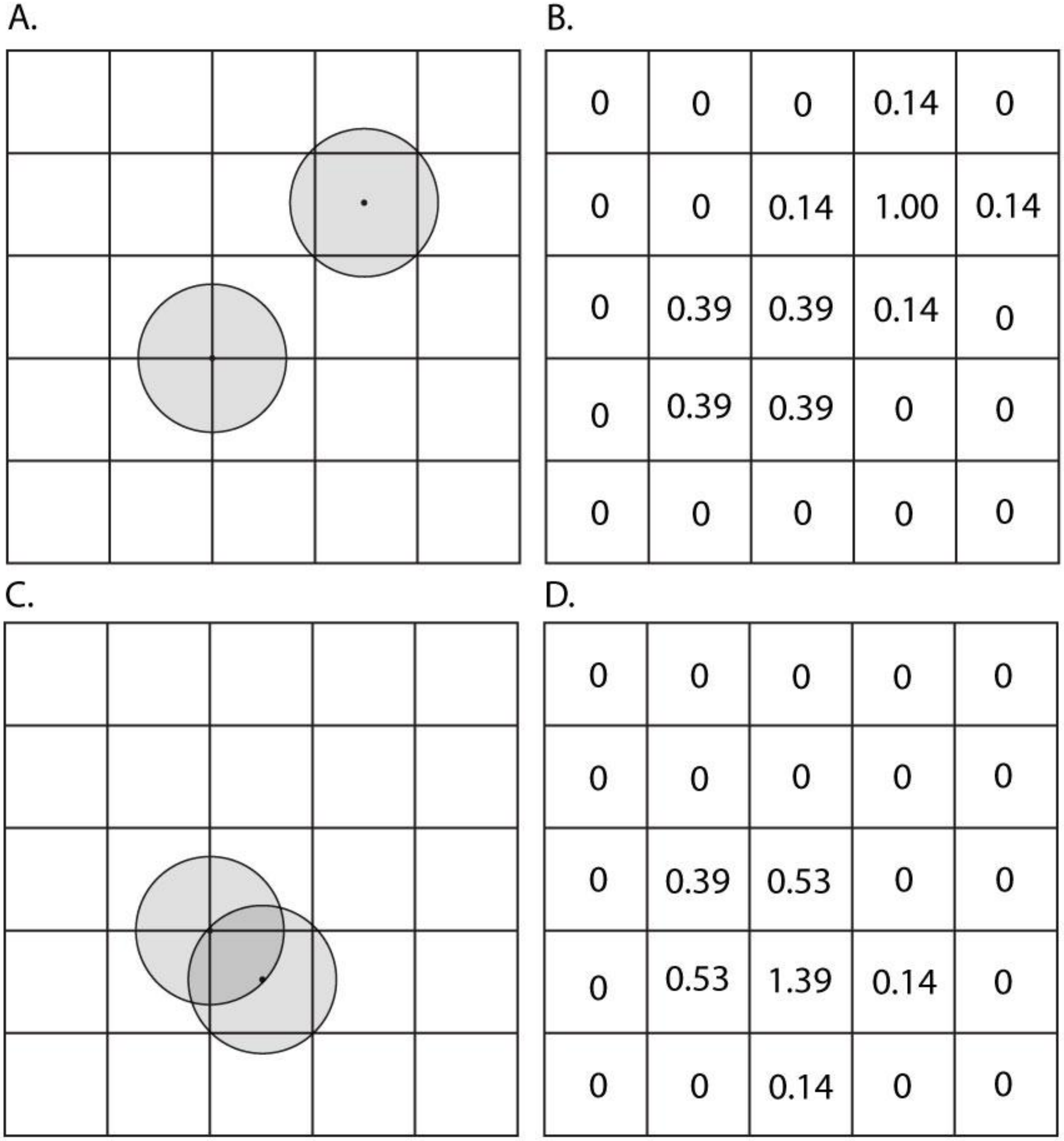

Figure S3. Calculation of annual grizzly bear density index. Visual representation of how the density index is calculated for a given year. Each grid cell is evaluated for occupancy by an activity range (grey circles in A and B) and a value corresponding to the amount of overlap is generated for each cell (B). For cells with more than one activity range (C) the value is the sum of proportional ranges within that cell (D).

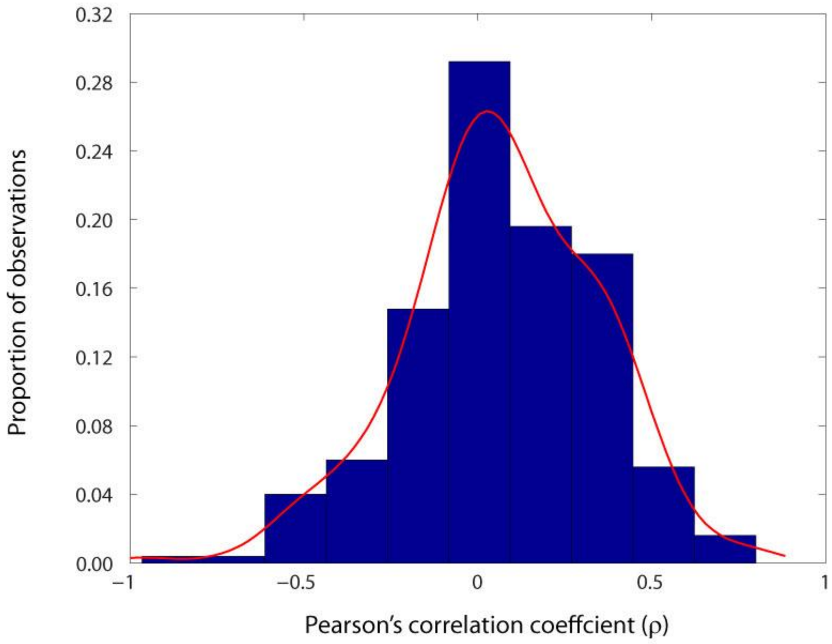

Figure S4. Correlation coefficients between grizzly bear density index and trapping effort. Distribution of correlation coefficients between the first derivatives of the grizzly bear density and trapping indices (n = 250). The red line represents a kernel density estimate.

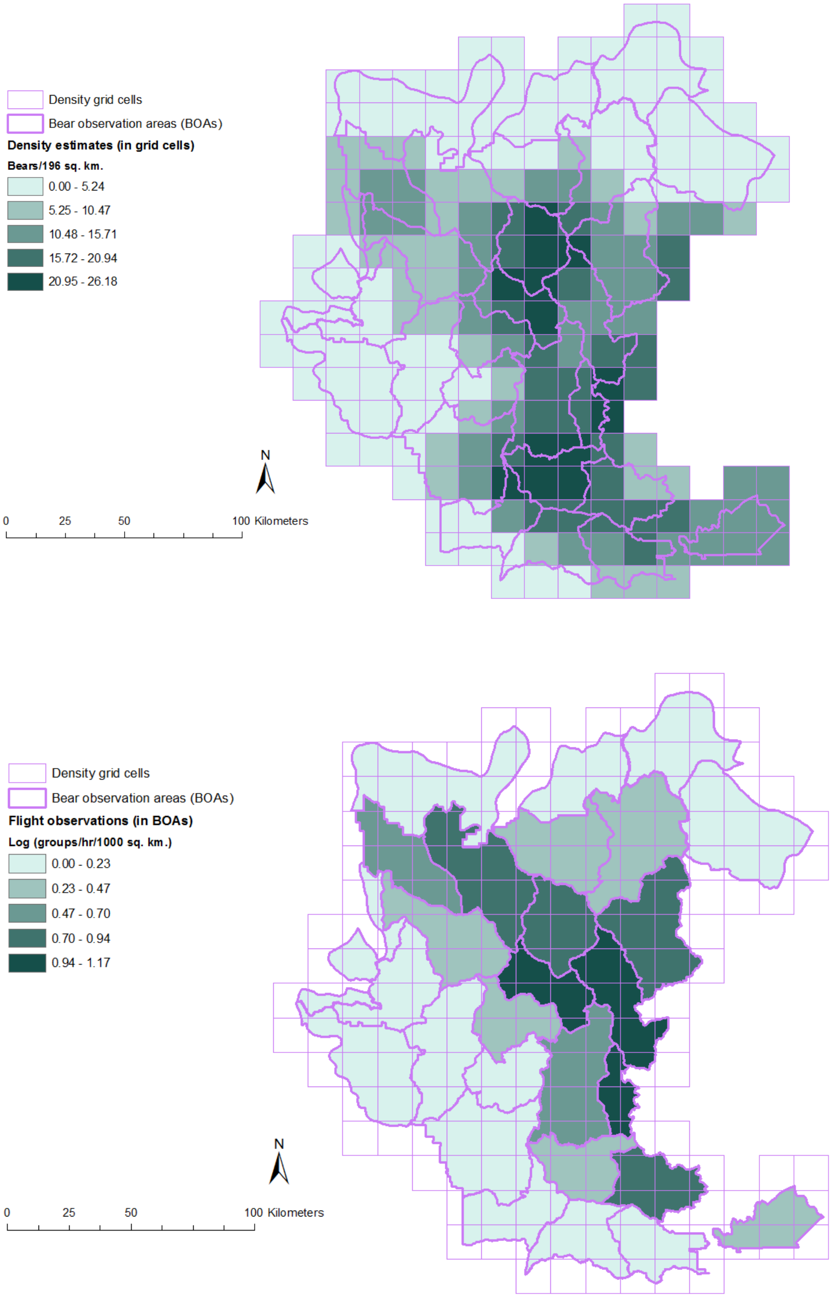

Figure S5. Grizzly bear density index and observation rate. Mean annual grizzly bear density index (no. cumulative activity ranges/196-km^2 grid cell) in the Greater Yellowstone Ecosystem during 1998–2006 (top) and median annual observation rate (log[bear groups/hr/1,000 km^2]) obtained during bear observations flights within bear observations areas (BOAs) during 1998–2006 (bottom).

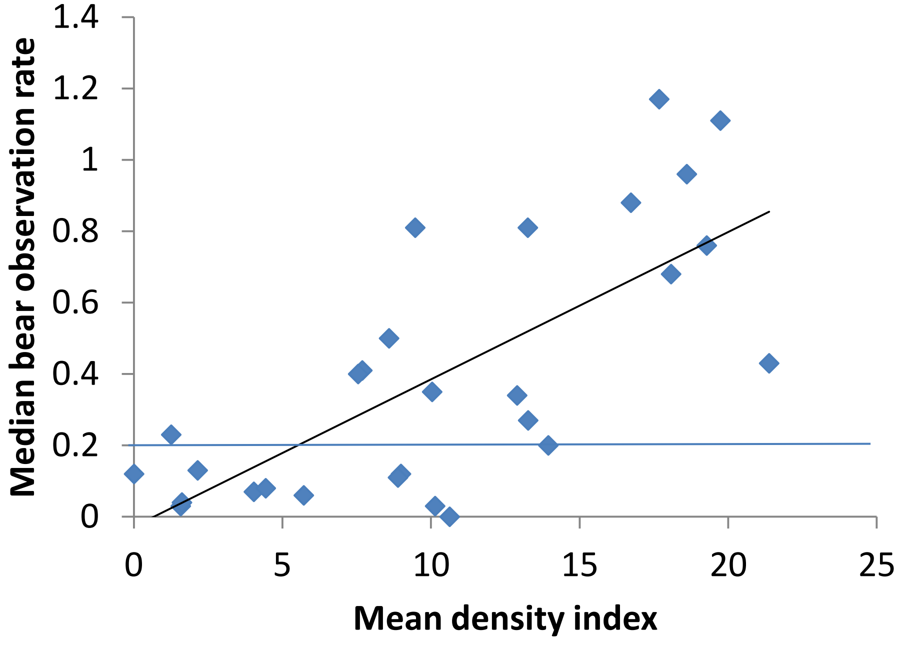

Figure S6. Relationship between mean grizzly bear density index and median observation rate. Relationship between mean annual grizzly bear density index (no. cumulative activity ranges/196-km^2 grid cell) and median annual grizzly bear observation rate from aerial surveys (log[no. bear groups/hr/1,000 km^2]) for 28 grizzly bear observation areas, Greater Yellowstone Ecosystem, 1998–2006.

References

1. Rotella JJ, Ratti JT (1986) Test of a critical density index assumption: a case study with gray partridge. Journal of Wildlife Management 50: 532–539.
2. Schwartz CC, Haroldson MA, White GC, Harris RB, Cherry S, et al. (2006) Temporal, spatial, and environmental influences on the demographics of grizzly bears in the Greater Yellowstone Ecosystem. Wildlife Monographs 161: 1–68. (doi:10.2193/0084-0173(2006)161[1:TSAEIO]2.0.CO;2)
3. Interagency Grizzly Bear Study Team (2012) Updating and evaluating approaches to estimate population size and sustainable mortality limits for grizzly bears in the Greater Yellowstone Ecosystem. Bozeman, MT: U.S. Geological Survey, Northern Rocky Mountain Science Center. 66 p.
4. Bjornlie DD, Thompson DJ, Haroldson MA, Schwartz CC, Gunther KA, et al. (2013) Methods to estimate distribution and range extent of grizzly bears in the Greater Yellowstone Ecosystem. Wildlife Society Bulletin (doi:10.1002/wsb.368)
